# From *M. chelonae* to *M. immunogenum*: A rare case of dual nontuberculous mycobacterial infection

**DOI:** 10.1016/j.jdcr.2025.04.036

**Published:** 2025-05-21

**Authors:** Ifeanyi Kingsley Uche, Nathalia Simonetti, Andrea Murina

**Affiliations:** a Louisiana State University Health Sciences Center, New Orleans, Louisiana; b Department of Dermatology, Tulane University School of Medicine, New Orleans, Louisiana

**Keywords:** immunosuppression, *Mycobacterium chelonae*, *Mycobacterium immunogenum*, nontuberculous mycobacterial infection

## Introduction

Cutaneous infections from nontuberculous mycobacteria (NTM) are increasing, especially in immunosuppressed patients.^1^ We report a case of an immunosuppressed female patient who initially developed a cutaneous *M. chelonae* infection on her left lower extremity, which healed following macrolide treatment. However, 1 month later, a new ulcer developed at a nearby site, which was subsequently identified as *M. immunogenum*. Our report highlights the importance for clinicians, particularly dermatologists, to maintain a high index of suspicion for atypical mycobacterial infections in immunosuppressed individuals.

## Case report

**Fig 1:**
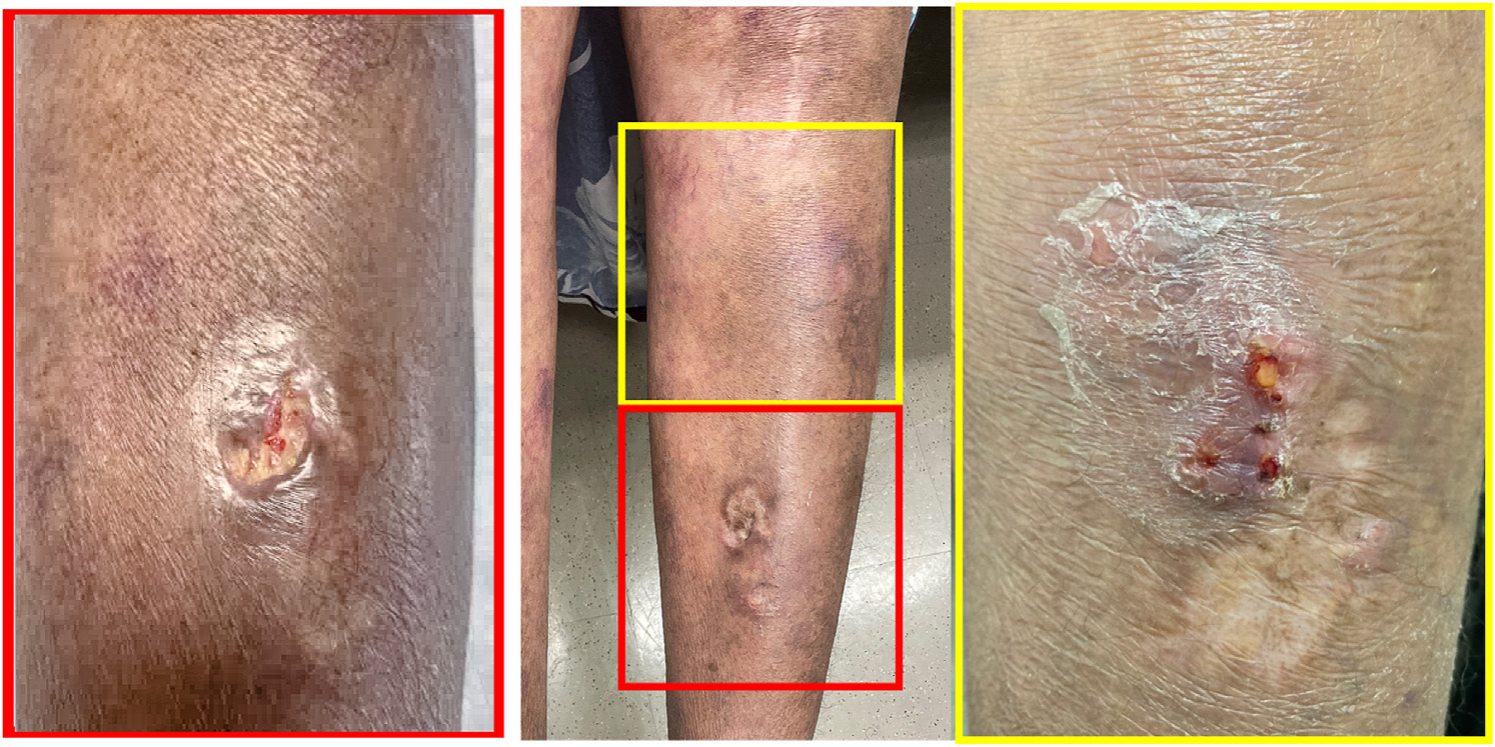
**(Left)** Initial left pretibial ulceration upon presentation. **(Center)** Complete resolution after clarithromycin treatment. **(Right)** Subsequent left pretibial ulceration.

A 46-year-old woman with a history of rheumatoid arthritis (treated with infliximab, methotrexate, and hydroxychloroquine), latent tuberculosis (treated 6 years prior), hypertension, and osteoporosis presented for evaluation of a left lower extremity pretibial ulceration. Physical examination of the left pretibial leg revealed a 1.5 cm ulcer with a violaceous border and overlying yellow heme-crusted fibrinous exudate (Fig 1). The patient was initially treated empirically with trimethoprim-sulfamethoxazole and levofloxacin. After the polymerase chain reaction results from the National Jewish Medical Center identified *M. chelonae*, the patient was transitioned to clarithromycin 500 mg twice daily for 6 months. 1 month after the resolution of her left lower extremity ulcer (Fig 1), a new ulcer developed superior to the previous ulcer (Fig 1), which was confirmed by polymerase chain reaction as *M. immunogenum*.

## Discussion

**Fig 2:**
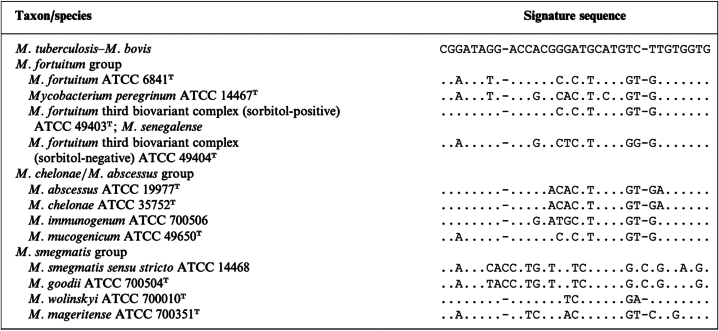
Signature nucleotides within hypervariable region A of the 16S rRNA gene of pathogenic rapidly growing mycobacteria. *M. tuberculosis* is used as the reference species. Dots indicate nucleotides identical to the *M. tuberculosis* sequence.

NTM are ubiquitous environmental opportunistic organisms that can cause infections in humans and are difficult to diagnose and treat. *M. immunogenum* is an NTM, first described in 2001 as a rapidly growing mycobacteria belonging to the *Mycobacterium chelonae*/*Mycobacterium abscessus* group.^2^ *M. immunogenum* closely resembles *M. chelonae* and *M. abscessus* and may have been misdiagnosed before the emergence of molecular identification techniques. *M. immunogenum* 16S rRNA sequence differs by 8 and 10 base pairs from *M. abscessus* and *M. chelonae,* respectively^2^ (Fig 2).

A high index of suspicion for atypical mycobacterial infections is essential in cutaneous diseases, especially in immunosuppressed patients. Accurate biopsy, culture, and identification testing are crucial, as *M. chelonae* and *M. immunogenum* are closely related, differing by only a few base pairs (Fig 2). It is possible that the initial testing did not differentiate between the 2 species. Treatment adequacy is important. NTM infections are usually treated for 6 months with antibiotics based on susceptibility. Of note, antimicrobial resistance is prevalent among NTM species. For example, *M. abscessus* harbors an erythromycin ribosomal methylase gene that confers resistance to macrolides, including clarithromycin and erythromycin, whereas *M. chelonae* lacks the erythromycin ribosomal methylase gene and is typically susceptible to clarithromycin.^3^ However, a recent report notes a case of M. *chelonae* exhibiting resistance to clarithromycin.^4^ *M. immunogenum* is susceptible to clarithromycin, amikacin, and linezolid.^5^, ^6^, ^7^ In our case, the patient was prescribed clarithromycin for 6 months. It is possible that the patient had concurrent *M. chelonae* and *M. immunogenum* infections. While rare, coinfections with multiple NTM species, particularly in immunocompromised individuals, have been reported.^8^

Environmental or host factors may have contributed to the development of the new ulcer. The patient sustained a traumatic wound to her left shin near the previous ulcer site, which later healed. It is possible that she was infected with *M. immunogenum* during this injury. Cutaneous *M. immunogenum* has been linked to localized infections following trauma, injections, or surgery,^9^ and is also more commonly seen in immunocompromised individuals.^5^

When taken together, accurate species identification and susceptibility testing are essential for selecting antimicrobial agents in a timely and informed manner. Furthermore, utilizing a dual antimicrobial regimen that demonstrates adequate bacterial susceptibility may reduce the likelihood of NTM species developing acquired resistance and decrease the incidence of recurrent infections.

## Conflicts of interest

Dr Murina is a speaker for AbbVie, Amgen, Bristol Meyers Squibb, Eli Lilly and Company, Janssen, Ortho-Dermatologics Pfizer, and UCB. She has served as a consultant for Bristol Meyers Squibb, Janssen, Novartis, Ortho-Dermatologics, Sun Pharma, Takeda, and UCB. Drs Uche, Simonetti, and Murina have no conflicts to declare.
